# Urgent dental care use in the North East and Cumbria: predicting repeat attendance

**DOI:** 10.1038/s41415-022-3886-6

**Published:** 2022-02-11

**Authors:** Charlotte Currie, Simon Stone, Mark Pearce, David Landes, Justin Durham

**Affiliations:** 4141590849001 School of Dental Sciences, Newcastle University, Newcastle Upon Tyne, UK; Newcastle upon Tyne Hospitals NHS Foundation Trust, Newcastle Upon Tyne, UK; 4141590849002 Population Health Sciences Institute, Newcastle University, Newcastle Upon Tyne, UK; 4141590849003 Public Health England, Newcastle Upon Tyne, UK

## Abstract

**Introduction** Around one-third of the UK population are 'problem-orientated dental attenders', only seeking care when suffering with dental pain and often on a repeated basis to secondary care. Little is known about attendance in primary care. The aim here was to examine the period prevalence of repeat urgent care attenders and establish predictors of repeat attendance in primary care.

**Methods** Data on urgent and emergency dental care attendances in primary dental care in the North East and Cumbria were analysed from 2013-2019. Variables included: patient sex; ten-year age band; lower super output area; and Index of Multiple Deprivation. Period prevalence was calculated and data were considered year by year to identify trends in attendances. Analysis was with descriptive statistics and predictors of repeat attendance were identified using logistic regression modelling.

**Results** Over the six-year period, there were 601,432 attendances for urgent primary dental care, equating to a period prevalence of 2.76% for the geographic population studied. In total, 16.15% of attendances were repeat attendances (period prevalence 0.45%) and predictors included being a woman and residence in deprived and rural areas. All urgent care attendances decreased over the six-year period, with one-off attendances beginning to increase again in 2019, while repeat attendances stabilised.

**Conclusion** Interventions to encourage regular dental attendances should be targeted at patients from the most deprived and rural areas of the North East and Cumbria; however, a decrease in repeat attendance was noted in these areas.

## Key points

- Predictors of being a repeat attender for urgent and emergency dental care included being a woman and living in the most deprived and rural areas of the North East and Cumbria.
- Over a six-year period (2013-2019), the number of one-off urgent and emergency dental care attenders to primary care in the North East and Cumbria decreased before beginning to increase.
- Over the same period, the number of repeat urgent and emergency dental care attenders to primary care in the North East and Cumbria decreased before stabilising.

## Introduction

Just under 10% of the dentate population in England, Wales and Northern Ireland report experiencing acute dental pain^1^ which is known to have a significant impact on everyday life.^2^^,^^3^ Despite this, almost one-third of the UK population are so called 'problem-orientated attenders',^1^^,^^4^^,^^5^ only seeking care when they have acute dental pain or problems, often waiting over two months before doing so.^6^^,^^7^^,^^8^ As well as affecting their quality of life, this also puts them at risk of serious adverse events such as unintentional paracetamol overdose^9^^,^^10^^,^^11^^,^^12^^,^^13^^,^^14^ and life-threatening infections.^15^^,^^16^^,^^17^^,^^18^ As problem-orientated attenders only seek care when they have acute dental pain, they frequently use drop-in services in secondary care, often on a repeated basis and for the same problem,^3^^,^^19^ as well as presenting to other healthcare professionals including hospital (medical) emergency departments,^20^^,^^21^^,^^22^ general medical practitioners^23^^,^^24^ and other allied health professionals.^25^^,^^26^^,^^27^^,^^28^ They will also seek urgent or emergency dental treatment with primary care general dental practitioners; however, little is known about the rates or predictors of repeat attendance in primary care. It is important that research is carried out to understand problem-orientated dental attendance so that interventions can be developed to encourage regular dental attendance and part of this understanding must include where these patients attend, to ensure that any interventions are sited in the appropriate places.

The North East and Cumbria covers a population of just under three million people, with a slight predominance of women at 51%.^29^ The North East of England has a slightly different demographic to that of Cumbria, with Cumbria having a generally older population and more rural areas.^30^ Access to dental services also varies between the North East and Cumbria, with 2-4% of North East residents reporting being unable to access dental care, compared to 8% of Cumbria.^31^ A further 12% of those responding to the National GP Survey stated that they did not try to access care because they thought that they would not be able to get an appointment.^31^ In addition, previous commissioning reports have shown that Cumbria has higher utilisation rates of urgent dental care services than the North East.^32^

The aim of this study was to determine the period prevalence of repeat urgent and emergency care attendance in the North East and Cumbria and identify any sociodemographic predictors of repeat attendance to inform intervention development aimed at problem-orientated dental attendance.

## Methods

A request was made to the NHS Business Service Authority for data available on Band 1 Urgent Course of Treatment FP17 claims during the period of April 2013 to April 2019 for Cumbria, Northumberland, Tyne and Wear and Durham, Darlington and Teesside legacy area teams. Data requested included: patient sex; ten-year age band; lower layer super output area (LSOA); and Index of Multiple Deprivation (IMD). To avoid disclosure of patient-identifiable information, the data were aggregated into the number of urgent care attendances before being made available to the authors for analysis. According to the UK's Human Research Authority's processes, the aggregated and anonymous data used within this paper did not mandate ethical review or approval.

IMD is the official measure of deprivation in the UK^33^ and considers deprivation being related to more than just poverty. IMD combines seven different domains: income; employment; health deprivation and disability; education, skills and training; crime; barriers to housing and services; and living environment. There are 32,844 LSOAs in England, with each being assigned a ranked IMD score, with 1 being the most deprived area and 32,844 being the least deprived. For the purposes of this study, IMD was considered in deciles and quintiles: quintile or decile 1 is the most deprived and quintile 5 or decile 10 the least deprived. IMD data were provided as part of the data request.

To take into account the variation in population sizes within the areas studied, the prevalence of urgent care attendances were calculated using freely available census data during the year of interest for the relevant population.^29^ The prevalence period was calculated as a percentage of the population registered on the census and therefore of all the population of interest who could theoretically access a dentist in that area. Population estimates were not used. LSOA was used for location-relevant outcomes including mapping the data to Office for National Statistics urban/rural definitions^34^ and also to middle layer super output area (MSOA) to allow mapping of the prevalence by area using the Public Health England Local Health Mapping Tool.^35^ A repeat urgent care user was defined as someone attending urgent care twice or more in one year, in order to capture data on frequent urgent care users and therefore most likely to represent problem-orientated dental attenders. Data were considered year by year to identify any changes in trends over the six-year period. These were analysed using descriptive statistics and univariate and multivariable logistic regression modelling with interaction and likelihood ratio analysis using STATA v15 (StataCorp LLC, College Station, TX, USA). Logistic regression modelling was repeated with adjustments for any potential confounders and included in the final model where a larger than 10% change was observed.

## Results

**Fig. 1:**
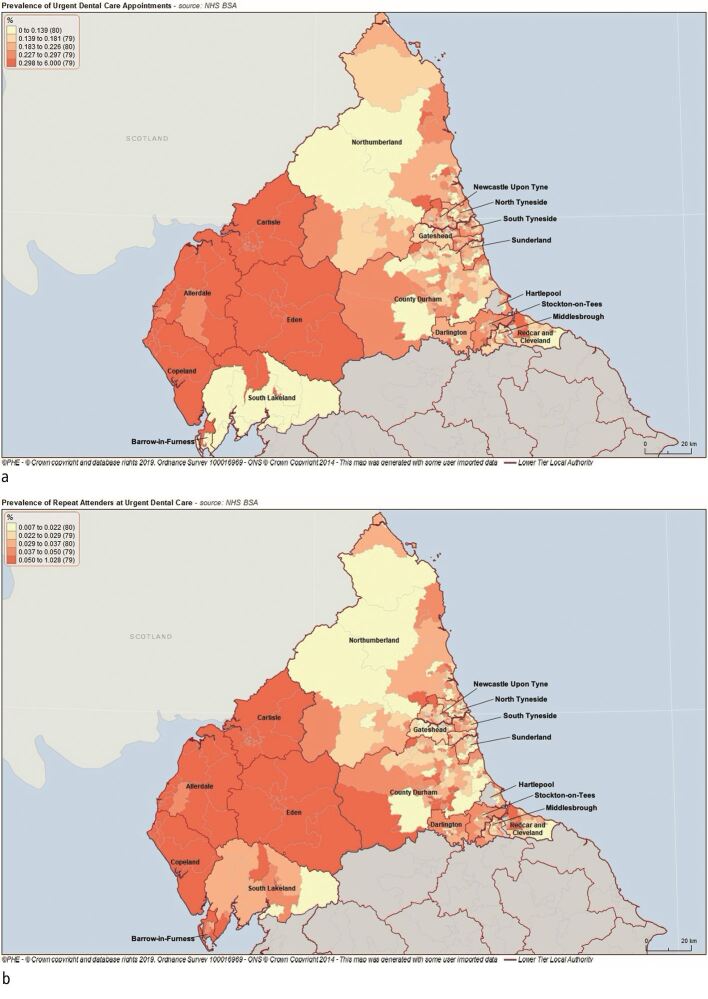
Urgent care attendances by MSOA. a) All urgent care attendances. b) Repeat urgent care attendances. Mapping software was obtained from www.localhealth.org.uk, Public Health England

**Fig. 2:**
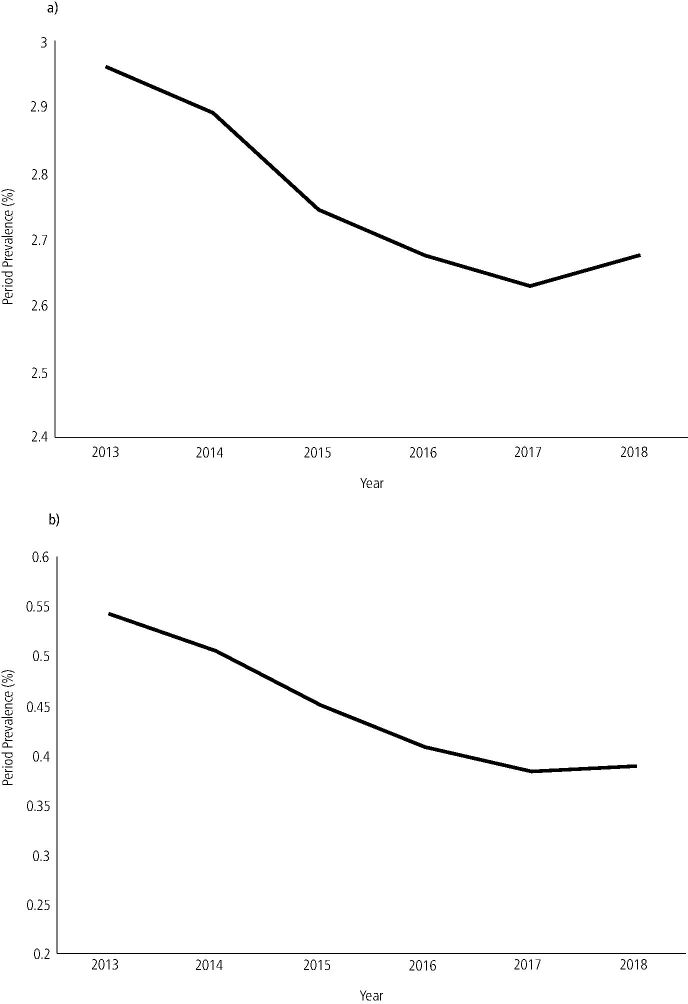
Number of attendances over the six-year period shown as prevalence to take in account changes in population size. a) All urgent care attendances. b) Repeat urgent care attendances

Over the six-year period there were 601,432 patient attendances for urgent and emergency dental care, which equates to an overall period prevalence of 2.76% for the North East and Cumbria population. When considered as a prevalence, the majority of these patients were women (population prevalence 3.3% women, 3.1% men), aged 30-39 years old and from most deprived areas of the North East (Table 1). Attendances increased in older age groups before decreasing from the seventh decade. The most common area for attendances was Copeland (Fig. 1). The majority of attendances were from rural locations (population prevalence 4.6% compared to 3.5% for non-rural locations). Attendances decreased from 2013-2017 and then began to increase again in 2018 (Fig. 2).

**Table 1:** Sociodemographic details of patients attending for urgent dental care

Demographic	All urgent care patients		Repeat urgent care patients	
	No. patients	Prevalence of population (%)	No. patients	Prevalence of population (%)
**Sex**				
Male	283,301	3.1	41,484	0.45
Female	318,131	3.3	55,671	0.58
**Age group (years)**				
0-9	48,844	2.32	6,652	0.32
10-19	56,242	2.71	6,781	0.33
20-29	78,450	3.19	13,807	0.56
30-39	77,940	3.58	13,573	0.62
40-49	84,944	3.47	14,978	0.61
50-59	90,983	3.42	16,362	0.61
60-69	81,241	3.54	13,576	0.59
70-79	55,615	3.48	8,056	0.50
80-89	23,792	2.93	2,996	0.37
90+	3,381	2.12	374	0.23
**IMD decile**				
1	100,051	3.41	18,530	0.63
2	82,860	3.38	14,092	0.57
3	73,044	3.32	12,276	0.56
4	63,435	3.13	10,211	0.50
5	58,265	3.03	8,871	0.46
6	42,608	3.10	6,652	0.47
7	47,059	3.10	6,891	0.45
8	45,066	2.96	6,521	0.42
9	50,772	3.07	7,472	0.45
10	35,530	3.11	5,071	0.44
**Area**				
Copeland	14,460	4.17	2,694	0.78
Allerdale	20,687	3.56	3,991	0.69
Carlisle	21,426	3.30	4,163	0.64
Eden	9,890	3.13	1,512	0.48
South Tyneside	33,081	1.32	5,061	0.20
Middlesbrough	30,094	1.29	5,922	0.25
Redcar and Cleveland	27,250	1.23	4,105	0.19
County Durham	108,181	1.22	19,131	0.22
Stockton on Tees	39,244	1.21	6,806	0.21
Darlington	22,157	1.20	3,843	0.21
Northumberland	64,769	1.18	10,652	0.19
North Tyneside	38,964	1.15	5,781	0.17
Sunderland	50,391	1.14	6,533	0.15
Newcastle Upon Tyne	53,417	1.11	8,526	0.18
Gateshead	35,180	1.06	4,637	0.14
Barrow-in-Furness	2,373	0.59	302	0.07
South Lakeland	2,245	0.36	225	0.04

The majority of patients attended for one urgent or emergency care appointment over the six-year period (83.9%), the remainder attending for more than one urgent or emergency care appointment. Repeat attenders accounted for 97,155 (16.15%) patient attendances over the six-year period, equating to an overall period prevalence of 0.45%. Patients who were repeat attenders tended to be women (0.58% compared to 0.45% prevalence), from the most deprived areas of the North East and aged 30-39 years old (Table 1). The prevalence of repeat attenders by year are shown in Figure 2, with a decrease seen from 2013-2017, before stabilising in 2018. Repeat attendances tended to be from rural areas (0.78% compared to 0.56% prevalence). The location of repeat attenders are shown in Figure 1.

**Fig. 3:**
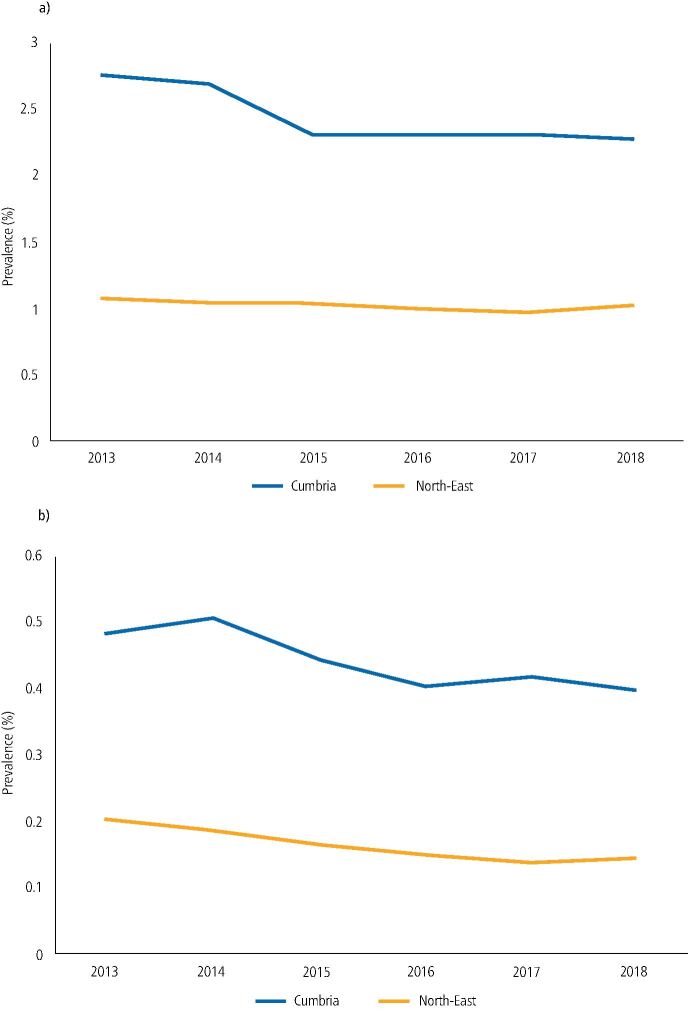
Number of attendances over the six-year period shown as a prevalence for the North East compared to Cumbria. a) All urgent care attendances. b) Repeat urgent care attendances

Given the difference in access to dental services between the North East and Cumbria, the prevalence between the two geographical areas was compared over time (Fig. 3). The prevalence of all and repeat patients attending for urgent dental care was consistently higher in Cumbria compared to the North East.

**Fig. 4:**
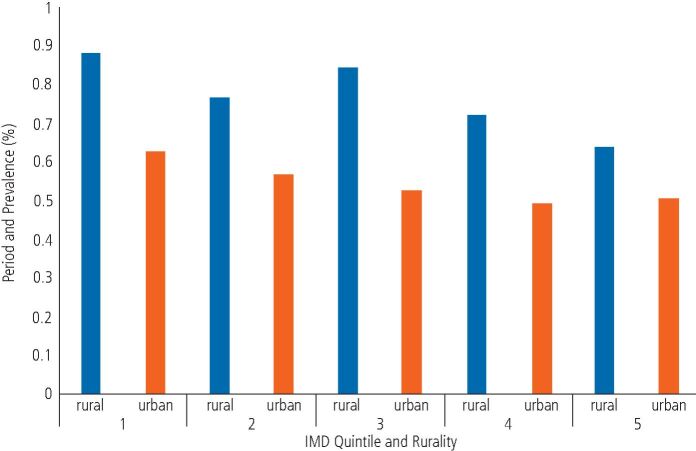
Relationship between IMD quintile and rurality for repeat attenders

Using univariate logistic regression modelling repeat attenders were less likely to be men (OR 0.8, 95% CI: 0.80-0.82, p <0.0001) and from urban areas of the North East and Cumbria (OR 0.9, 95% CI: 0.90-0.95, p <0.0001). In addition, repeat attenders were more likely to be from more deprived areas (OR 0.93, 95% CI: 0.93-0.94, p <0.001) (Table 2). Within multivariable regression modelling, a significant interaction was found between being a repeat attender and IMD quintile and rurality (p <0.00001). The relationship between IMD quintile and rurality for repeat attenders is shown in Figure 4, whereby repeat attenders are less likely to be from the least deprived and urban areas of the North East (OR 0.89, 95% CI: 0.83-0.95, p <0.0001).

**Table 2:** Odds ratios and 95% confidence intervals for logistic regression modelling of repeat attenders by IMD quintile (all p values <0.0001). IMD quintile 1 (most deprived) is used as a comparator, therefore showing that those living in areas of IMD quintile 2 are almost 10% less likely to be a repeat attender than those in quintile 1. Patients living in IMD quintile 3 are over 15% less likely to be a repeat attender than those in quintile 1 and patients in IMD quintiles 4 and 5 are over 20% less likely to be a repeat attender than those in quintile 1

IMD quintile	Odds ratio	95% CI
1	Comparator	Comparator
2	0.91	0.89-0.93
3	0.84	0.82-0.86
4	0.78	0.77-0.80
5	0.78	0.77-0.80

**Fig. 5:**
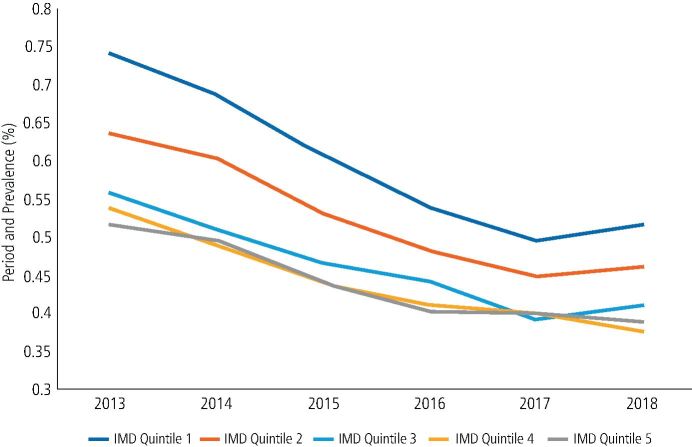
Number of repeat attenders over time by IMD quintile

Considering IMD quintile over time, people from the most deprived areas of the North East remained the majority of repeat attenders. The overall number of repeat attenders in each quintile decreased from 2013-2017, however from 2017 the number of repeat attenders in quintiles 1-3 increased, while those in quintiles 4-5 continued to decrease (Fig. 5).

## Discussion

Over a six-year period in the North East and Cumbria, the period prevalence of all urgent and emergency dental care attendances in primary dental care was 2.76%. In total, 16.5% of these attendances were repeat attendances, which equated to a 0.45% period prevalence. This is a lower repeat attendance rate than observed in secondary care where around one-third of attenders are repeat attenders.^3^^,^^8^ The majority of the patients attending were in their fourth decade and from the most deprived areas of the North East and Cumbria which is in keeping with the typical sociodemographic of patients attending secondary care urgent dental clinics^3^ and medical emergency departments^20^ in the same region, as well as nationally^8^ and internationally.^6^^,^^36^^,^^37^^,^^38^^,^^39^^,^^40^^,^^41^^,^^42^^,^^43^^,^^44^^,^^45^^,^^46^^,^^47^^,^^48^^,^^49^^,^^50^ However, in contrast to the demographic attending secondary urgent care services the majority of patients were women. This may be because female patients are more likely to attend for routine dental care^4^ and as such, be undergoing an active course of treatment at the practice, making access for urgent care easier in comparison to those who are not undergoing active treatment. Unfortunately, a limitation to this study is that it is unknown what proportion of the patients attending for urgent and emergency dental care were undergoing an active course of treatment and therefore may reflect those attending with complications associated with treatment, rather than from avoiding regular dental care.

Predictors of being a repeat attender reflected the typical sociodemographic of all attendees which included being a woman from rural and deprived areas. The odds of being a repeat attender varied in relation to deprivation depending on their urban or rural status, with those having the highest odds for repeat attendance living in the most deprived and rural areas. Patients from deprived areas may be more likely to seek repeat urgent and emergency care due to an increase in prevalence of dental disease and pain,^1^^,^^51^ fewer seeking regular preventive dental care^4^ and having poorer health literacy.^52^ Living in a rural area is also associated with a decreased likelihood of attending for regular preventive care^53^^,^^54^^,^^55^^,^^56^ which may be partly explained by patients reporting oral health as a low priority,^56^ in addition to dental access potentially being more challenging, which is known to be a problem in Cumbria compared to the North-East^32^ and may explain the difference in attendances between the two geographical areas observed.

Attending primary dental care services in a problem-orientated manner means that patients are more likely to continue to suffer with oral health problems^1^^,^^57^^,^^58^ and fail to receive standard preventive dental care.^57^ This continues to put them at risk of adverse health events as well as exert a direct and indirect economic impact on the patient and wider society. For this reason, it is imperative that interventions are developed to try and encourage regular preventive dental care over and above problem-orientated dental care. In primary dental care in the North East and Cumbria, these interventions should therefore be targeted to patients residing in the most deprived and rural areas to ensure those who would benefit the most receive them. Although the current literature has been used to provide some explanation as to why these particular patient groups may be repeat attenders, the data analysis cannot provide casual evidence for the reasons behind repeat attendance. This warrants further research exploring the specific barriers within these patient groups.

Changes in attendance patterns were noted over the time period studied, with a decrease in attendance noted from 2013-2017 and repeat attendance remaining stable into 2018, while one-off urgent care attendance began to increase. In addition, all and repeat urgent care attendances were consistently higher in Cumbria than the North East. This could indicate that either service improvements or interventions aimed at repeated urgent and emergency dental attendance in primary care may need to be prioritised in Cumbria. Whereas in the North East, interventions could be sited in other clinical settings where these patients are more likely to attend, such as secondary care urgent dental care clinics.^3^ The reasons why problem-orientated attenders chose to present repeatedly to secondary care rather than primary care are under-researched; however, could include cost of service and availability of immediate walk-in treatment. Changes in attendance patterns by IMD were also noted over the six-year period, with an increase in repeat attenders from the more deprived quintiles of the North East and a decrease from the least deprived quintiles, indicating a potential increase in oral health inequalities across the region.

It should be noted that the findings of this study are limited to attendees at urgent and emergency dental care before the COVID-19 pandemic, which has had a significant impact on dental care internationally. At the start of the pandemic in March 2020, all routine dental care ceased in the UK and patients were only able to access urgent and emergency dental care in dedicated hubs.^59^ As the pandemic progressed, access to dental care subsequently improved with individual practices offering urgent and emergency care before transitioning to offer a mix of urgent and more routine dental care. Therefore, the majority of the UK population will have changed their attendance habits. At this stage, it is uncertain what long-term impact there will be on engagement with routine dental care and as a result, the proportion of problem-orientated attenders could increase and this will warrant further future research. In addition, this study examines part of the UK where access to dental care in Cumbria is known to be an issue with an increase in urgent dental care attendance. Findings may therefore be affected by these access issues and may not be representative of the rest of the UK. Further work is required in other areas to establish if predictors of repeat urgent dental care attendance is comparable elsewhere. This dataset also covered NHS dental care only and therefore may not represent patients accessing private dental care.

## Conclusion

In conclusion, across the North East and Cumbria during a six-year period, there were 601,432 patient attendances for urgent and emergency dental care, equating to an overall period prevalence of 2.76%. To put this another way, nearly 3 in every 100 people in the region need urgent care. Repeat attenders were more likely to be women and from the most deprived and rural areas; however, the prevalence of repeat attendance declined over the study period. Any interventions developed to promote regular dental care should therefore be targeted at patients residing in the most deprived and rural areas of the region.
